# Single Nucleotide Polymorphisms in the *HIRA* Gene Affect Litter Size in Small Tail Han Sheep

**DOI:** 10.3390/ani8050071

**Published:** 2018-05-04

**Authors:** Mei Zhou, Zhangyuan Pan, Xiaohan Cao, Xiaofei Guo, Xiaoyun He, Qing Sun, Ran Di, Wenping Hu, Xiangyu Wang, Xiaosheng Zhang, Jinlong Zhang, Chunyuan Zhang, Qiuyue Liu, Mingxing Chu

**Affiliations:** 1 Key Laboratory of Animal Genetics and Breeding and Reproduction of Ministry of Agriculture, Institute of Animal Science, Chinese Academy of Agricultural Sciences, Beijing 100193, China; 1229zhoumei@163.com (M.Z.); pzq170450077@163.com (Z.P.); caoxiaohan2007@hotmail.com (X.C.); guoxfnongda@163.com (X.G.); hedayun@sina.cn (X.H.); sunqing0514@sina.com (Q.S.); dirangirl@163.com (R.D.); pinkyhoho@163.com (W.H.); xiangyu_wiggle@163.com (X.W.); 2 College of Agriculture and Forestry Science, Linyi University, Linyi 276000, China; 3 College of Life Science, Sichuan Agricultural University, Ya’an 625014, China; 4 Tianjin Institute of Animal Sciences, Tianjin 300381, China; zhangxs0221@126.com (X.Z.); jlzhang1010@163.com (J.Z.); 5 State Key Laboratory for Agrobiotechnology, College of Biological Sciences, China Agricultural University, Beijing 100193, China; dachunyuanzi@cau.edu.cn; 6 Beijing Advanced Innovation Center for Food Nutrition and Human Health, China Agricultural University, Beijing 100193, China

**Keywords:** WGS, SNPs, *HIRA* gene, expression, fecundity

## Abstract

**Simple Summary:**

Litter size is one of the most important reproductive traits in sheep. Two single nucleotide polymorphisms (SNPs), g.71874104G>A and g.71833755T>C, in the Histone Cell Cycle Regulator (*HIRA*) gene, were identified by whole-genome sequencing (WGS) and may be correlated with litter size in sheep. The two SNPs were genotyped and expression patterns of *HIRA* was determined in sheep breeds with different fecundity and in groups of Small Tail Han sheep producing large or small litters. Association analysis indicated that both SNPs were significantly correlated with litter size in Small Tail Han sheep. Furthermore, high levels of *HIRA* expression may have a negative effect on litter size in Small Tail Han sheep.

**Abstract:**

Maintenance of appropriate levels of fecundity is critical for efficient sheep production. Opportunities to increase sheep litter size include identifying single gene mutations with major effects on ovulation rate and litter size. Whole-genome sequencing (WGS) data of 89 Chinese domestic sheep from nine different geographical locations and ten Australian sheep were analyzed to detect new polymorphisms affecting litter size. Comparative genomic analysis of sheep with contrasting litter size detected a novel set of candidate genes. Two SNPs, g.71874104G>A and g.71833755T>C, were genotyped in 760 Small Tail Han sheep and analyzed for association with litter size. The two SNPs were significantly associated with litter size, being in strong linkage disequilibrium in the region 71.80–71.87 Mb. This haplotype block contains one gene that may affect litter size, Histone Cell Cycle Regulator (*HIRA*). *HIRA* mRNA levels in sheep with different lambing ability were significantly higher in ovaries of Small Tail Han sheep (high fecundity) than in Sunite sheep (low fecundity). Moreover, the expression levels of *HIRA* in eight tissues of uniparous Small Tail Han sheep were significantly higher than in multiparous Small Tail Han sheep (*p* < 0.05). *HIRA* SNPs significantly affect litter size in sheep and are useful as genetic markers for litter size.

## 1. Introduction

China has great diversity in its sheep resources. Most indigenous Chinese sheep breeds are uniparous and only a few breeds have two or more offspring. Litter size is one of the most important reproductive traits in sheep, which is affected by genes with minor effects [1,2]. Whole-genome sequencing (WGS) is now widely used to identify genetic variants underlying adaptive traits. By sequencing the genomes of individuals from representative populations many genetic variations contributing to phenotypic diversity in various species have been successfully characterized [3,4,5]. Population structure and genome selection among Chinese indigenous sheep breeds have been investigated using single nucleotide polymorphism (SNP) screening assays [6,7].

Histone Cell Cycle Regulator (*HIRA*) is a histone chaperone, which maps to sheep chromosome 17 and consists of 27 exons and codes for 1091 amino acids. *HIRA* is involved in various chromatin regulatory processes, such as gene transcription, sperm chromatin remodeling and embryonic development [8,9,10]. Until now, most *HIRA* studies focused on embryogenesis in fish and mice [11]. *HIRA* is reported to be crucial for H3.3 variant deposition during chromatin assembly and the depletion of it can affect H3.3/H4 replacement, which affects mouse oocyte development by preventing the oocytes from maintaining the full dynamic range of gene expression [12,13]. In murine embryogenesis, *HIRA* inactivation can lead to embryonic lethality mid-gestation [14]. Nashun et al. [15] reported that *HIRA* was critical for transcriptional regulation and DNA methylation in the process of mouse oogenesis, and depletion of *HIRA* in primordial oocytes can lead to severe developmental defects associated with extensive oocyte death, which is consistent with a previous study by Veselovska et al [16]. Moreover, a point mutation in *HIRA* can lead to female infertility in *Drosophila*; mutant female *Drosophila* eggs prevent the decondensation of sperm nuclei after fertilization so that the paternal chromosome cannot participate in development [17,18]. Further study in vertebrates showed that *HIRA* mainly participates in the processes of fertilization or embryonic development, but has no significant effect on oogenesis or meiosis [19].

To date, some major genes affecting litter size in sheep have already been identified, such as bone morphogenetic protein receptor 1B (*BMPR1B*), bone morphogenetic protein 15 (*BMP15*) and growth differentiation factor 9 (*GDF9*), which all belong to the transforming growth factor β (TGF-β) superfamily and map to sheep chromosomes 6, X and 5 respectively, and mutations in each of these genes can affect the ovulation rate of ewes [20,21,22]. Since *BMPR1B* has been identified as the major gene affecting fertility in Booroola Merino Sheep, Davis et al. [23] validated that the presence of *BMPR1B* can significantly affect litter size of Small Tail Han a Hu sheep from China as well, and FecB mutation of BMPR1B had an additive effect on ovulation rate in these sheep breeds. Later, a strong evidence of selection at the *BMPR1B* locus in highly prolific breeds Hu and Large-tail Han sheep was identified by Wei et al. [7] based on the Illumina Ovine SNP50 Genotyping BeadChip. *BMP15* was essential for the early development of follicle in sheep. Eight mutations (*FecX^I^*, *FecX^H^*, *FecX^G^*, *FecX^B^*, *FecX^L^*, *FecX^R^*, *FecX^Gr^*, *FecX^O^*) related with fertility have been identified in sheep. All those heterozygotes had the higher fertility than wild type but homozygotes were sterile except *FecX^Gr^* and *FecX^O^* [24,25,26,27,28,29]. *GDF9* have been reported to be associated with litter size in Araucana creole sheep [1], Salsk and Volgograd sheep [30]. Like most mutations of *BMP15*, the heterozygotes of *GDF9* had the highest fertility.

In the previous study of our laboratory, WGS was performed on 89 Chinese domestic sheep from nine different geographic locations (Small Tail Han, Hu, Cele black, Ujimqin, Bayinbuluke, Tan, Oula, Prairie Tibetan and Valley Tibetan sheep) and ten Australian sheep [31] to detect new polymorphisms affecting horn phenotype. In addition, those WGS data was also used to identify new polymorphisms affecting plateau hypoxia adaptability, seasonal estrus and litter size in sheep by our group (unpublished data). Chinese domestic sheep breeds were divided into two groups of high fecundity (Small Tail Han, Hu and Cele black sheep) and low fecundity (Ujimqin, Bayinbuluke, Tan, Oula, Prairie Tibetan and Valley Tibetan sheep). The selective sweep assay [32,33] was adopted to analyze the degree of genetic differentiation between the two groups. *HIRA* was positionally identified as a candidate gene for litter size according to the fixation index (*F*_ST_) value [34]. Two SNPs, g.71874104G>A and g.71833755T>C, in *HIRA* were genotyped. The expression pattern of *HIRA* and its association with litter size in sheep was investigated based on whole genome data. Our study aimed to reveal whether *HIRA* was the major gene affecting litter size in sheep.

## 2. Materials and Methods

All the experimental procedures mentioned in the present study were approved by the Science Research Department (in charge of animal welfare issue) of the Institute of Animal Sciences, Chinese Academy of Agricultural Sciences (IAS-CAAS) (Beijing, China). Ethical approval on animal survival was given by the animal ethics committee of IAS-CAAS (No. IASCAAS-AE-03, 12 December 2016).

### 2.1. WGS and Detection of Single Nucleotide Polymorphisms

We previously sequenced 89 Chinese domestic sheep from nine different geographic locations and ten Australian Merino sheep, the coverage depth after genome alignment was approximately six-fold for each individual, resulting in more than 50× coverage depth for each breed [31]. To detect selective sweeps in Chinese domestic sheep with different fecundity, *F*_ST_ statistics were calculated with a 50-kb sliding window with a step of 25 kb on the genome based on *F*_ST_ between two groups of sheep, a high fecundity group (Small Tail Han, Hu and Cele black sheep) and a low fecundity group (Ujimqin, Bayinbuluke, Tan, Oula, Prairie Tibetan and Valley Tibetan sheep). Raw sequence data has been submitted to the NCBI Sequence Read Archive (SRA) under accession number SRP066883 [31]. SNPs were called using SAMtools^51^ (version 1.2) [35] after pooling all samples from the same breed and the removal of low-quality SNPs. Then the *F*_ST_ was normalized using the formula Z*F_ST_* = |*x_A_*−*μ_x_*|/*σ_x_*, where *x_A_* represents the *F*_ST_ value, *μ_x_* and *σ_x_* represent the mean and standard deviation of the *x_A_*, and Z*F*_ST_ > 5 was regarded as a significant selective sweep region [31].

The standardized pooled heterozygosity, Z*_H_*, and allele frequency differences, Δ_AF_, between the two groups were calculated to support the sweep signals. The pooled heterozygosity *H* was calculated with a 20-kb sliding window and a 10-kb step using the formula *H* = 2∑*p*∑*q*/(∑*p* + ∑*q*)^2^, where ∑*p* represents the sum of the major allele frequencies of all SNP sites in the window and ∑*q* represents the sum of the minor allele frequencies. The standardized heterozygosity, Z*_H_*, was calculated as Z*_H_* =|*H* − *μ_H_*|/*σ_H_*, where *μ_H_* and *σ_H_* denote the mean and standard deviation of *H* [31]. Δ_AF_ was calculated for the biallelic SNP sites by measuring the differences in the reference allele proportions between groups. The employed thresholds for Z*_H_* and Δ_AF_ were both top 5% at genome-wide level.

### 2.2. Selection of Animals and DNA Preparation

As detailed in Table 1, 760 ewes from six sheep breeds were selected for genotyping, in order to verify whether the results of WGS could be applied to other Chinese sheep breeds with different fecundity.

Small Tail Han sheep is a famous breed in China which was notable for its high prolificacy with mean litter size of 2.61. It also has excellent performance on meat and fur. The number of Small Tail Han sheep was about 5–8 million in 2010 in our country, and it mainly distributes at southwest of Shandong Province, northeast of Henan Province, south of Hebei Province and north of Anhui and Jiangsu Province [36]. Jugular vein blood samples (10 mL blood per ewe) were collected using citrate glucose as anticoagulant. Genomic DNA was extracted by the phenol-chloroform method [37], and then dissolved in ddH_2_O. Genotyping of each sample was performed using 20 μL DNA at 40–80 ng/μL. Reagents and instruments for genotyping were all from Beijing Genenode Biotech Co., Ltd. (Beijing, China).

### 2.3. Primer Design

The primers for genotyping were designed using Agena software from Beijing Genenode Biotech Co., Ltd. (Beijing, China) according to the sheep *HIRA* sequences (GenBank accession no. NC_019474.1). qPCR primers were designed using primer 3 software [38] and sheep *HIRA* mRNA (GenBank accession no. XM_015101463.1) and *β*-actin mRNA sequences (GenBank accession no. NM_001009784). All primers were synthesized by Beijing Tianyihuiyuan Biotechnology Co. Ltd. (Beijing, China) Primer information is listed in Table 2.

### 2.4. Genotyping

The g.71874104G>A and g.71833755T>C loci in the *HIRA* gene were selected for genotyping in 760 samples from Small Tail Han, Tan, Sunite, Suffolk, Dorper, and Prairie Tibetan sheep. Genotyping was performed using PCR and primer extension and mass spectrometric analysis (iPlex assay, Sequenom, San Diego, CA, USA) on a Sequenom MassArray according to the manufacturer’s instructions (http://www.sequenom.com). Polymerase chain reactions were carried out in 5 µL containing 1.0 µL 20–50 ng/µL genomic DNA, 0.5 µL 10 × PCR buffer, 0.4 µL 25 mmol/L MgCl_2_, 0.1 µL 25 µmol/L dNTP, 1.0 µL PCR Primer mix, 0.2 µL *Taq* DNA polymerase (Promega, Madison, WI, USA), and ddH_2_O. PCR conditions were as follows: initial denaturation at 95 °C for 2 min, followed by 45 cycles of denaturation at 95 °C for 30 s, annealing at 56 °C for 30 s, extension at 72 °C for 60 s, with a final extension at 72 °C for 10 min. Primer extension reactions were carried out in 2 µL containing 0.2 µL iplex Buffer, 0.2 µL Terminator mix, 0.94 µL Extend primer mix, 0.04 µL iplex Enzyme, and ddH_2_O. Extension conditions were as follows: initial denaturation at 94 °C for 30 s, followed by 40 cycles of denaturation at 94 °C for 5 s, annealing at 52 °C for 5 s, with a final extension at 72 °C for 3 min. Only those samples with a >95% success rate and only those SNPs with a genotype success rate of >95% were included in the analysis.

### 2.5. RNA Extraction, cDNA Synthesis and qPCR 

Three ewes under each category (high fecundity Small Tail Han vs. low fecundity Sunite sheep; multiparous vs. uniparous Small Tail Han sheep) were selected for expression study. RNA was extracted from eight tissues (brain, cerebellum, hypothalamus, pituitary, ovary, oviduct, uterus body and uterine horn) which are especially vital for mammal reproduction using the RNAprep pure Tissue Kit (Tiangen, Beijing, China) according to the kit instructions with the minor modification of using Trizol (Invitrogen, Carlsbad, CA, USA) as the lysis solution. Quantity and quality of total RNA were determined using a Nanodrop2000 and 1.2% agarose gel electrophoresis. RNAs were stored at −80 °C until use. cDNAs were synthetized by reverse transcription (PrimeScript™ RT reagent kit; TaKaRa) carried out in 20 μL containing 1000 ng RNA, 4 μL 5 × PrimeScript Buffer, 1 μL PrimeScript RT Enzyme Mix I, 1 μL Oligo dT primer 1 μL Random 6 mers and RNase free ddH_2_O. The conditions were as follows: 37 °C 15 min, 85 °C 5 s. cDNA samples were diluted five-fold and stored at −20 °C. qPCR was performed on a Roche Light Cycler^®^ 480II (Roche, Beijing) and carried out in 20 μL containing 10 μL SYBR Premix Ex Taq II (TaKaRa, Dalian), 0.8 μL each primer, 2 μL cDNA and 6.4 μL ddH_2_O. The conditions were as follows: initial denaturation at 95 °C for 5 min, followed by 40 cycles of denaturation at 95 °C for 5 s, annealing at 60 °C for 30 s. *β*-actin was used as a reference gene.

### 2.6. Statistics

Genotype and allele frequency, *PIC*, *HE* and *NE* were calculated. Then, the distribution of genotypes for each SNP in the studied population were tested for deviation from Hardy–Weinberg equilibrium by the Hardy–Weinberg law [30,39]. Statistical analyses were performed using SAS (V. 8.1) (SAS Institute Inc., Cary, NC, USA). Differences among three groups of samples were tested by the least significant difference test. *P* values less than 0.05 were considered to be significant. The adjusted linear model was: *y_ijn_* = *μ*+ *P_i_* + *G_j_* + *I_PG_* + *e_ijn_*, where *y_ijn_* is the phenotypic value of litter size; *μ* is the population mean; *P_i_* is the fixed effect of the *i*th parity (*i* = 1, 2, 3); *G_j_* is the fixed effect of the *j*th genotype (*j* = 1, 2, 3); *I_PG_* is the interaction effect of parity and genotype; and *e_ijn_* is the random residual. To assess the litter size between genotypes and parity in sheep, the correlation was calculated using scaled imputed log-transformed litter size values. The gene expression data were normalized to the reference gene *β*-actin and relative expression level was calculated by the 2^−ΔΔ*C*T^ method.

## 3. Results

### 3.1. Genotype Analysis of Variants Located in the Significant Sweep Region

**Figure 1:**
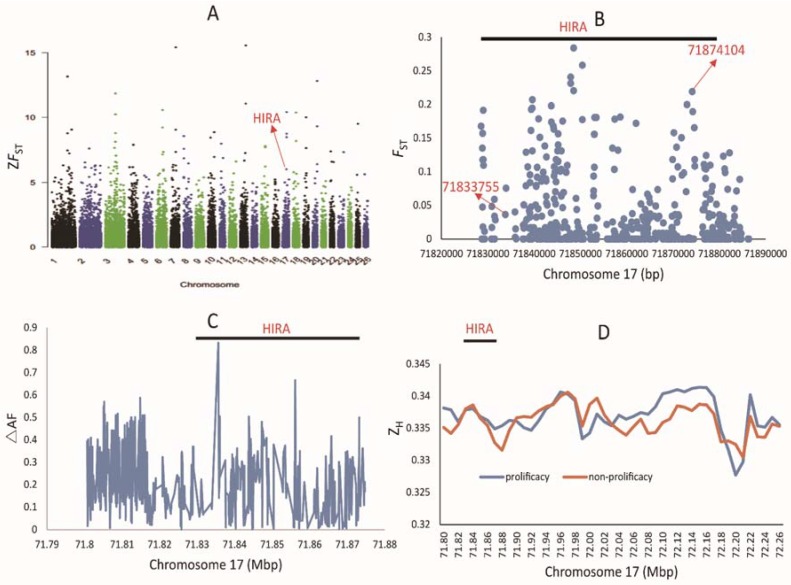
Manhattan plots of genome-wide Z*F*_ST_ and signals of selective sweeps in the region 71.80–71.87 Mb. (**A**). Manhattan plots of genome-wide Z*F*_ST_ and the positive selected genes; (**B**). The Scatter plot of *F*_ST_ value in selected region near 71.80–71.87 Mb; (**C**). Δ_AF_ value between fecundity and non-fecundity sheep in the region near 71.80–71.87 Mb; (**D**). Z*_H_* value between fecundity and non-fecundity sheep in the region near 71.80–71.87 Mb.

WGS revealed that 38,090,348 SNPs in total were screened and the windows with top five percent of values were considered as the significant threshold for single statistic. In total, we obtained 153 positive sweep regions. The selective sweep region (71.80–71.87 Mb) was under a slightly selection (Z*F*_ST_ = 7.05) which only includes *HIRA* gene, but there were no significant differences in Z*_H_* and Δ_AF_ of this region (Figure 1C,D, see File 1 and File 3 in Supplementary Materials for details). After gene annotation of selective sweep regions, we found that *HIRA* gene played a key role in fertilization of mouse [19], while the genes in other selected regions were related to highland adaptability, seasonal estrus and hair color. We did choose *HIRA* gene for further study. All SNPs near this region of *HIRA* gene were checked, and only two SNPs, g.71874104G>A (strongest selection in upstream region) and g.71833755T>C (missense mutation of *HIRA*), were under strong linkage disequilibrium in this region (see File 2 in Supplementary Materials for details). Those two SNPs were chosen for subsequent research. The Manhattan plots of genome-wide Z*F*_ST_, the Scatter plot of *F*_ST_ value in selected region 71.80–71.87 Mb, Z*_H_* and Δ_AF_ value of the same region were all shown in Figure 1.

Genotyping results of g.71874104G>A and g.71833755T>C revealed that GG, GA and AA genotypes existed at the g.71874104G>A locus, while TT, CT and CC existed at the g.71833755T>C locus. The dominant genotype and allele were GA and A, respectively, for g.71874104G>A; while for g.71833755T>C, the dominant genotype and allele were TT and T, respectively.

### 3.2. Population Genetic Analysis of Polymorphism in the HIRA Gene

Population genetic analyses of the two loci in six sheep breeds were performed. The results are shown in Table 3. The results revealed that the g.71874104G>A locus was moderately polymorphic (0.25 < *PIC* < 0.5) in all sheep breeds (Table 3). The g.71833755T>C locus was moderately polymorphic in Small Tail Han, Tan and Prairie Tibetan sheep, and at a low rate of polymorphism (*PIC* < 0.25) in Sunite, Suffolk and Dorper sheep (Table 3). The chi-square test indicated that g.71874104G>A was under Hardy-Weinberg equilibrium (*p* > 0.05) while g.71833755T>C was not (Table 3).

### 3.3. Association Analysis of Two Polymorphisms with Litter Size in Small Tail Han Sheep

Association analysis between the polymorphic loci, g.71874104G>A and g.71833755T>C and the litter size of Small Tail Han sheep was performed. The least squares mean and standard error for litter size of each parity and the average litter size of three parities were shown in Table 4. These data indicate that g.71874104G>A was significantly correlated with the litter size of the third parity of Small Tail Han sheep, and the litter size of the GA genotype was the highest for each parity; the g.71833755T>C locus was significantly correlated with the litter size of the second parity, the third parity and the average litter size of the three parities (*p* < 0.05), genotype CT had the highest litter size for each parity.

### 3.4. Expression of HIRA in Sheep with Different Fecundity and in Small Tail Han Sheep with Different Litter Size

**Figure 2:**
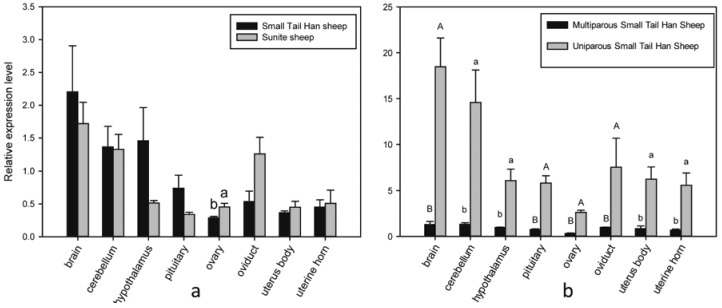
Expression of *HIRA* in reproductive tissues of sheep. (**a**) Expression of *HIRA* in reproductive tissues of high fecundity Small Tail Han sheep and low fecundity Sunite sheep; (**b**) Expression of *HIRA* in reproductive tissues of multiparous and uniparous Small Tail Han sheep. Note: Different small letters in the same group mean significant difference (*p* < 0.05), different capital letters in the same group mean highly significant difference (*p* < 0.01).

The expression of *HIRA* in eight reproductive tissues of high fecundity Small Tail Han sheep and low fecundity Sunite sheep is shown in Figure 2a. Figure 2a indicates that *HIRA* is expressed in all tissues with the highest level in the brain, followed by cerebellum, hypothalamus and oviduct. The expression of *HIRA* in the ovary, oviduct, uterus body and uterine horn of Small Tail Han sheep was higher than that in Sunite sheep, but in the other tissues, expression was lower in Small Tail Han sheep compared with that in Sunite sheep. However, expression of *HIRA* was not significantly different in any tissue between the two breeds (*p* > 0.05) except for the ovary (*p* < 0.05).

The expression of *HIRA* in multiparous and uniparous Small Tail Han sheep is shown in Figure 2b. Figure 2b indicates that *HIRA* is expressed most highly in the brain, followed by cerebellum and oviduct. The expression level of *HIRA* in uniparous Small Tail Han sheep was significantly higher than that in multiparous Small Tail Han sheep in all tissues (*p* < 0.05).

## 4. Discussion

In the present study, one significant sweep region was located in the genomic region, 71.80–71.87 Mb, by selective sweep analysis based on WGS data. Two SNPs, g.71874104G>A and g.71833755T>C, were in strong linkage disequilibrium in this region and this haplotype block was mapped to chromosome 17 and in the genomic region of the *HIRA* gene. These results indicated that this region is important in the study of litter size in sheep. There were no reported quantitative trait loci (QTLs) associated with reproductive traits in this region; only two QTLs were reported in or near this region, and both of them were related to behavior traits (https://www.animalgenome.org/cgi-bin/QTLdb/OA/index). Here, two SNPs, g.71874104G>A and g.71833755T>C, were genotyped and the expression patterns of *HIRA* in sheep breeds with different fecundity or within Small Tail Han sheep with different litter size were characterized.

The present study indicates that g.71874104G>A has a strong potential for selection in all sheep breeds examined, while g.71833755T>C only has a strong potential for selection in Small Tail Han, Tan and Prairie Tibetan sheep. We, therefore, concluded that the selection intensity of different loci in the same sheep breeds and the same locus in different sheep breeds were both different. The chi-square test showed that g.71874104G>A has a genetic advantage in adaptability after long-term evolution and selection; while g.71833755T>C was not like g.71874104G>A in most of the breeds we examined. There were no mutations at the g.71833755T>C locus in Dorper sheep, indicating that larger sample sizes are needed in future research.

Association analysis revealed that mutations at g.71874104G>A and g.71833755T>C had a great effect on litter size in Small Tail Han sheep, which is consistent with the linkage disequilibrium result. The g.71874104G>A locus is 255 bp upstream of the transcription start site of *HIRA* and is, therefore, probably located in the gene’s promoter, the most important region in a gene for regulating transcriptional efficiency. In addition, in silico prediction (https://www-bimas.cit.nih.gov/molbio/signal/) identified a CAC-binding_pro-transcription factor binding site 10 bp from the g.71874104G>A locus. CAC-binding_pro transcription factor is an enhancer-binding protein that recognizes CCACC. Considering the above two findings, we suggest that variation at the g.71874104G>A locus may influence transcription efficiency by changing binding activity of the CAC-binding_pro transcription factor. g.71833755T>C is a missense mutation site, which caused the amino acid at this position to change from a hydrophilic glutamine to an alkaline arginine. We also suggest that variation at the g.71874104G>A or g.71833755T>C loci may affect the deposition of histone into nucleosomes, which can also affect transcriptional efficiency [40,41,42].

The *HIRA* expression in ovary, oviduct and uterus of low fecundity Sunite sheep was higher than those in high fecundity Small Tail Han sheep indicated that the expression of *HIRA* may negatively regulate the function of these organs which were especially important for mammal reproduction. Further, it was reported that the hypothalamic-pituitary-gonadal axis (HPG) is the most important system controlling the secretion of the sex hormones gonadotropin-releasing hormone (GnRH), follicle-stimulating hormone (FSH) and luteinizing hormone (LH) in mammals [43,44]. GnRH, FSH and LH are the most important reproductive hormones controlling the reproductive activities of mammals. In our study, the expression level of *HIRA* in the HPG of uniparous Small Tail Han sheep was significantly higher than that in multiparous sheep, indicating that *HIRA* may have a negative effect on litter size of Small Tail Han sheep by regulating the secretion of sex hormones. These results further confirmed that *HIRA* is closely associated with litter size in Small Tail Han sheep. However, the sample size of each category for detecting *HIRA* expression in our study was still too low to get solid conclusion, larger sample size is needed to validate our result in the future.

From previous studies, we know that *HIRA* is essential for embryonic development in vertebrates and mutation of *HIRA* can lead to embryonic lethality [10,45]. *HIRA* plays a particular role in fertilization of *Drosophila* because it is a conserved replication-independent chromatin assembly factor that is essential for the assembly of paternal chromatin at fertilization [46]. Maternal *HIRA* is necessary for mouse development past the zygote stage by participating in the incorporation of histone variant H3.3 into the paternal genome, as in *Drosophila* [47,48]. Deletion of *HIRA* prevents nucleosome assembly in the mouse sperm genome, halting male pronucleus formation, thereby causing fertilization failure [49]. Further studies showed that the mutant female mouse could ovulate normally but was infertile when mated to a wild-type male, and that the litter size of wild-type and heterozygotes was not significantly different [47]. While it was interesting to find that the majority of fertilized embryos from heterozygous oocytes develop to blastocysts, no mutant zygotes progress to the two-cell stage [47]. In our study, mutations in the *HIRA* SNPs, g.71874104G>A and g.71833755T>C, did not cause infertility in ewes. However, heterozygotes for both g.71874104G>A and g.71833755T>C had the highest litter size in Small Tail Han sheep, indicating that heterozygotes may have the strongest developmental ability. From our results combined with those of previous reports, we speculated that the differences in litter size between sheep with different g.71874104G>A and g.71833755T>C genotypes may be caused by different developmental abilities of the oocytes produced.

## 5. Conclusions

Our study indicates that two SNPs, g.71874104G>A and g.71833755T>C, are highly associated with litter size in sheep based on WGS data and that *HIRA* is the only candidate gene in this significant sweep region. We considered that the expression level of *HIRA* may have a negative effect on litter size in Small Tail Han sheep, and this preliminary result needs to be validated in further studies employing a large sample size. Our study might provide a theoretical basis for breeding prolific sheep by molecular selection.

## Figures and Tables

**Table 1:** Information of six sheep breeds selected for genotyping.

Breed	Number	Type	District
Small Tail Han sheep	380	multiparous	Farmers home, Yuncheng, Shandong Province, China
Tan sheep	80	uniparous	Yanchi, Ningxia Hui Autonomous Region, China
Sunite sheep	100	uniparous	Wulatezhongqi, Bayannaoer, Inner Mongolia Autonomous Region, China
Suffolk sheep	39	uniparous	Beijing Aoxin Stud Farm Co. Ltd. located in Shunyi District, Beijing, China
Dorper sheep	30	uniparous	Beijing Aoxin Stud Farm Co. Ltd. located in Shunyi District, Beijing, China
Prairie Tibetan sheep	131	uniparous	Dangxiong, Tibet Autonomous Region, China

**Table 2:** *HIRA* primer information.

Primer Name	Primer Sequence	Product Size	Usage
*HIRA-1-F*	5′-ACGTTGGATGAAACGAAACCAGAGCTCTCC-3′	133 bp	Polymerase Chain Reaction (PCR) for g.71874104G>A
*HIRA-1-R*	5′-ACGTTGGATGTGTGCAGAGGGTCTGATAAC-3′		
*HIRA-1-E*	5′-GGGCCCGGCAACCGAGTTAGTC-3′		Extension reaction
*HIRA-2-F*	5′-ACGTTGGATGAGCCCGTGGGAAGACACTGT-3′	126 bp	PCR for g.71833755T>C
*HIRA-2-R*	5′-ACGTTGGATGAAGGCTTTCTGACAGTCCTC-3′		
*HIRA-2-E*	5′-CAGACCCGAGTAGAGCTCCCCT-3′		Extension reaction
*HIRA-3-F*	5′-CTGAGCGAGGAGGAGAAGAG-3′	108 bp	qPCR
*HIRA-3-R*	5′-CATCTCGGGGTTCTCGATGA-3′		
*β-actin-F*	5′-GCTGTATTCCCCTCCATCGT-3′	97 bp	qPCR
*β-actin-R*	5′-GGATACCTCTCTTGCTCTGG-3′		

**Table 3:** Population genetic analysis of g.71874104G>A and g.71833755T>C in six sheep breeds.

Locus	Breed	Genotype Frequency			Allele Frequency		*PIC*	*HE*	*NE*	*Chi*-Square Test (*p*-Value)
g.71874104G>A		GG	GA	AA	G	A				
	Small Tail Han sheep	0.11 (42)	0.45 (167)	0.44 (166)	0.33	0.67	0.35	0.45	1.80	1.00
	Tan sheep	0.16 (13)	0.46 (37)	0.38 (30)	0.39	0.61	0.36	0.48	1.91	0.78
	Sunite sheep	0.06 (6)	0.36 (36)	0.58 (57)	0.24	0.76	0.30	0.37	1.58	0.92
	Suffolk sheep	0.11 (4)	0.34 (13)	0.55 (21)	0.28	0.72	0.32	0.40	1.67	0.37
	Dorper sheep	0.18 (5)	0.32 (9)	0.50 (14)	0.34	0.66	0.35	0.45	1.81	0.13
	Prairie Tibetan sheep	0.10 (13)	0.37 (48)	0.53 (69)	0.28	0.72	0.32	0.41	1.69	0.29
g.71833755T>C		TT	CT	CC	T	C				
	Small Tail Han sheep	0.58 (215)	0.34 (126)	0.09 (32)	0.75	0.25	0.30	0.37	1.60	0.03
	Tan sheep	0.64 (51)	0.28 (22)	0.08 (7)	0.78	0.22	0.29	0.35	1.54	0.06
	Sunite sheep	0.72 (69)	0.22 (21)	0.06 (6)	0.83	0.17	0.24	0.28	1.40	0.02
	Suffolk sheep	0.92 (36)	0.08 (3)	0 (0)	0.96	0.04	0.07	0.07	1.08	0.80
	Dorper sheep	1 (30)	0 (0)	0 (0)	1.00	0.00	0.00	0.00	1.00	
	Prairie Tibetan sheep	0.74 (95)	0.17 (22)	0.09 (12)	0.82	0.18	0.25	0.29	1.41	0.00

Note: *PIC*, *HE* and *NE* represents polymorphic information content, heterozygosity and effective number of alleles, respectively; numbers in the bracket represent the number of detected sheep of each genotype; *p* > 0.05 indicates the locus was under Hardy-Weinberg equilibrium.

**Table 4:** Least squares means and standard error of litter size in Small Tail Han Sheep with different g.71874104G>A and g.71833755T>C genotypes.

Locus	Genotype	Litter Size of the First Parity	Litter Size of the Second Parity	Litter Size of the Third Parity	Average Litter Size
g.71874104G>A	GG	1.97 ± 0.13 (35)	2.24 ± 0.13 (41)	2.83 ± 0.29 ^b^ (7)	2.25 ± 0.13 (83)
	GA	2.19 ± 0.06 (154)	2.39 ± 0.07 (163)	3.09 ± 0.13 ^a^ (64)	2.51 ± 0.05 (381)
	AA	2.11 ± 0.06 (141)	2.11 ± 0.07 (159)	2.71 ± 0.13 ^b^ (54)	2.34 ± 0.05 (354)
g.71833755T>C	TT	2.16 ± 0.06 (193)	2.13 ± 0.06 ^b^ (211)	2.77 ± 0.11 ^b^ (78)	2.35 ± 0.04 ^b^ (466)
	CT	2.13 ± 0.07 (115)	2.45 ± 0.08 ^a^ (124)	3.16 ± 0.17 ^a^ (37)	2.56 ± 0.06 ^a^ (277)
	CC	2.04 ± 0.15 (27)	2.28 ± 0.15 ^ab^ (32)	2.67 ± 0.29 ^b^ (12)	2.29 ± 0.12 ^ab^ (68)

Note: Numbers in the parentheses next to litter size represent the amount of sheep of each genotype; Different small letters in the same group mean significant difference (*p* < 0.05).

## Supplementary Materials

The following are available online at http://www.mdpi.com/2076-2615/8/5/71/s1, File 1: Allele difference, File 2: *F*_ST_, File 3: Z*_H_*.
